# Field study evaluating the efficacy of a combination formulation of fluralaner with moxidectin and pyrantel (BRAVECTO^®^ TriUNO) against canine intestinal nematode infections

**DOI:** 10.1186/s13071-026-07348-7

**Published:** 2026-04-02

**Authors:** Rafael Chiummo, Eva Zschiesche, Zvezdelina Kirkova, Domenico Otranto, Marcos Bezerra-Santos, Elias Papadopoulos, Dhimiter Rapti, Lea Heinau

**Affiliations:** 1 MSD Saúde Animal, São Paulo, Brazil; 2 MSD Animal Health Innovation GmbH, Schwabenheim, Germany; 3 Faculty of Veterinary Medicine, Trakia University, 6000 Stara Zagora, Bulgaria; 4 Department of Veterinary Medicine, University of Bari, Valenzano, 70010 Bari, Italy; 5 Department of Veterinary Clinical Sciences, Jockey Club College of Veterinary Medicine and Life Sciences, City University of Hong Kong, Kowloon Tong Hong Kong, China; 6 School of Veterinary Medicine, Aristotle University, 54124 Thessaloniki, Greece; 7 Faculty of Veterinary Medicine, Agricultural University of Tirana, Tirana, Albania

**Keywords:** Ascarid, Canine, *Capillaria*, Fluralaner, Hookworm, Moxidectin, *Toxascaris*, *Toxocara*, *Trichuris*, Whipworm

## Abstract

**Background:**

There is a demand for broad-spectrum products effective against internal and external parasites to simplify dog-owner compliance with veterinary parasiticide treatment recommendations. A field study investigated the safety and efficacy of a broad-spectrum formulation of fluralaner, moxidectin, and pyrantel pamoate compared with a licensed afoxolaner–milbemycin oxime combination product in treating canine intestinal nematode infections in client-owned dogs at multiple sites in Europe.

**Methods:**

Dogs with positive pre-treatment fecal egg counts (FEC) were randomized to treatment on day 0 with the investigative veterinary product (IVP), at dose rates of: IVP, fluralaner 10–20 mg/kg, moxidectin 0.025–0.050 mg/kg, pyrantel 5–10 mg/kg (*n* = 172); or a control product (CP), afoxolaner 2.5–5.2 mg/kg, milbemycin oxime 0.5–1.0 mg/kg (*n* = 86). On day 14, FEC were completed on all study dogs. Efficacy against any intestinal nematode species was claimed if the FEC reduction in ≥ 10 dogs initially positive for that species was ≥ 90%, and if the geometric mean difference between pre- and post-treatment FEC was statistically significant (*P* ≤ 0.05).

**Results:**

Study dogs were enrolled at veterinary clinics in Albania (*n* = 60), Bulgaria (*n* = 111), Greece (*n* = 27), and Italy (*n* = 60). Where a sufficient number of infected dogs allowed for statistical comparison, geometric mean FEC reductions on day 14 versus day 0 in the IVP group were significant (*P* < 0.0001) for hookworms, *Toxocara canis*, *Toxascaris leonina, Trichuris vulpis*, and capillarid-type eggs. On the basis of geometric mean reductions in FEC relative to day 0, the efficacy of the IVP exceeded 99% against all intestinal nematode parasites, and FEC reductions in the CP exceeded 98%. The percentage of nematode-free dogs in the IVP group was statistically noninferior (*P* < 0.0001) to the CP group and even statistically superior at the *α* = 0.025 level of significance (one-sided). There were no treatment-related adverse events.

**Conclusions:**

In client-owned dogs, the combination of fluralaner–moxidectin–pyrantel is safe and effective for the treatment of intestinal infections with hookworms, ascarids, including *T. canis*, and whipworms. On the basis of the complete elimination of parasite ova from the feces evaluated in this study, this fluralaner–moxidectin–pyrantel combination demonstrated to be significantly superior (*P* < 0.025) to an afoxolaner–milbemycin combination in the treatment of canine intestinal nematode infections.

**Graphical Abstract:**

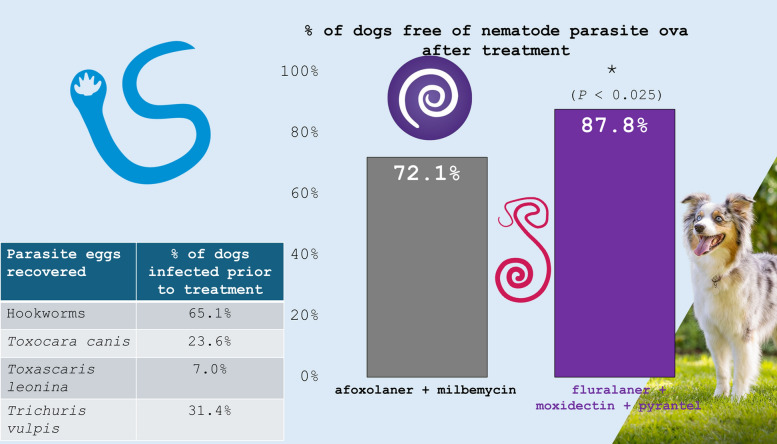

## Background

Despite the availability of effective anthelmintics, intestinal nematode parasites of dogs continue to be a problem throughout the world, presenting a risk to canine health and having zoonotic potential. Control of these parasites is dependent upon owner compliance to veterinary expert recommendations on the type and frequency of required treatments and the need to correctly dispose of their dog’s feces [1, 2]. Compliance failures can often be attributed to a lack of owners’ and practicing veterinarians’ awareness of those recommendations and a misunderstanding of which products treat which parasites (e.g., confusing an orally administered ectoparasiticide with similarly packaged endoparasiticides) [3–5]. Therefore, to simplify treatments and improve dog-owner compliance with expert recommendations, there is a need for broad-spectrum products that are effective against internal and external parasites.

To meet that demand, a broad-spectrum fluralaner-containing formulation with a combination of moxidectin and pyrantel pamoate, prepared as a chewable tablet, has been developed. The rationale for the fluralaner dose (10 mg/kg) in the formulation is to provide a full month’s efficacy against ectoparasites, as this orally administered dose is currently indicated in many countries as a monovalent fluralaner formulation (BRAVECTO^®^ 1-Month; MSD Animal Health) [6]. The safety of a 25 mg/kg dose of fluralaner has been established from 10 years of field experience as a market-leading ectoparasiticide (at that dose providing up to 3 months efficacy against ticks, fleas, and mites) [7].

Milbemycin oxime and moxidectin are macrocyclic lactones (ML) that have been available for more than 25 years, with their main indication being the prevention of canine heartworm disease. Both are effective against larvae of *Dirofilaria immitis* and adult *Angiostrongylus vasorum,* and have shown efficacy against intestinal nematodes [8–10]. Combination products with milbemycin oxime (minimum dose 0.5 or 0.75 mg/kg) with endo- and ecto-parasiticidal activity are currently approved for use in dogs in the EU [11, 12]. Moxidectin has been shown to be effective in preventing canine heartworm disease when administered orally at a dose of 0.003 mg/kg [13] and topically at a dose of 2.5 mg/kg against both heartworm and intestinal nematode infections [14]. Most recently, orally administered endectocidal combination products containing moxidectin have been approved for use in dogs at minimum doses of 0.020–0.024 mg/kg [15, 16]. On the basis of that moxidectin history, the minimum dose selected for this investigative formulation was 0.025 mg/kg. The third active ingredient in this formulation, pyrantel pamoate, has more than 50 years of use in humans and animals, demonstrating its safety and efficacy in the treatment of intestinal nematode infections.

The fluralaner–moxidectin–pyrantel combination formulation has been demonstrated in laboratory studies as effective in preventing angiostrongylosis [17] as well as treating canine infections with *A. vasorum* and adult intestinal nematodes (*Ancylostoma caninum* and *Uncinaria stenocephala*) [17, 18]. The field study design herein aimed to confirm that this formulation would be safe and effective in treating canine intestinal nematode infections in client-owned dogs at multiple sites in Albania, Bulgaria, Greece, and Italy.

## Methods

The study objective was to evaluate the efficacy of an investigative veterinary product (IVP) containing fluralaner (12.5% w/w), moxidectin (0.03%), and pyrantel (6.25%) (BRAVECTO® TriUNO, MSD Animal Health) against natural infections with intestinal nematodes. The efficacy of the IVP was compared with a commercially available positive control product (CP) containing afoxolaner and milbemycin oxime (NexGard Spectra^®^, Boehringer Ingelheim).

The study was Good Clinical Practice (GCP)-compliant [19], multicenter, positive-controlled, randomized, and investigator-masked, conducted from January through July 2022, and in accordance with the following guidelines: the World Association for the Advancement of Veterinary Parasitology (WAAVP) [20]; the diagnosis of helminth infections in humans and animals; the efficacy of anthelmintics, general requirements (VICH7)[21]; and statistical principles in veterinary clinical trials [22]. Prior to any study activities, the investigator at each site reviewed the protocol with the owner, including instructions to observe their dog for any adverse events (AE) and report accordingly. Each owner (or agent) then signed an informed consent.

The planned number of animals to determine efficacy was 150 dogs in the IVP group and 75 dogs in the CP group. With an estimated dropout rate of approximately 10%, the target number of animals to be included was 170 dogs in the IVP group and 85 in the CP group. This sample size had been set in accordance with the current anthelmintic evaluation guidelines [21].

On day 0, each dog was thoroughly examined by a veterinarian to determine suitability for inclusion in the study. The individual involved in treatment assignments and treatment administration to animals was not masked during the study and was not involved in clinical assessments. The investigator making clinical observations and the parasitologists responsible for fecal sample analysis were masked to treatment assignments.

Feces were collected twice: pre-treatment (no more than 7 days prior to the study treatment) and 14 days (± 2) after treatment. Feces were collected by study personnel or by the dog’s owner either at home, in the animal shelter, from voided samples, or directly from the rectum. Immediately after collection, samples were placed in refrigerated containers with cool pads to maintain a temperature of approximately 4–8 °C. On the day of collection or the following day, samples were forwarded to the designated diagnostic laboratory on the same day. At the laboratory, fecal egg identification and counting were done within 24 h (no more than 72 h) of sampling, with counting performed by Mini-FLOTAC® with saturated sodium chloride flotation solution and a sensitivity of five eggs per gram (EPG) [23]. Upon confirmation of a positive fecal test, a blood sample (approximately 1 mL) was collected to test for the presence of antigen of *D. immitis* and *A. vasorum* (SNAP Heartworm® RT Test for *D. immitis* antigen, IDEXX GmbH, Kornwestheim, Germany; for *A. vasorum,* AngioDetect™, IDEXX GmbH, Kornwestheim, Germany) A second fecal sample of enrolled dogs was collected for processing on day 14.

### Inclusion criteria

Clinically healthy dogs were enrolled into the study on the basis of the following criteria: age ≥ 8 weeks and body weight of ≥ 2 kg; confirmation by positive fecal sample of infection with at least one intestinal nematode species (i.e., hookworms, *Toxocara canis*, *Toxascaris leonina*, *Trichuris vulpis*, and others) (EPG > 0); and antigen test negative for *D. immitis* and *A. vasorum*. Dogs were excluded if treated with anthelmintics within 14 days before study start; an endectocide had been administered within 30 days or treated with fluralaner (BRAVECTO®) within 3 months of study start; if pregnant or lactating; or if having a history of epilepsy. Dogs with chronic medical conditions were included at the discretion of the enrolling veterinarian.

### Treatments

Upon enrollment, dogs were randomly assigned to one of the two study groups (IVP and CP) in a 2:1 ratio using site-specific computer-generated randomization lists. Dogs were treated once, on day 0, with either IVP or CP at the following dose rates: 10–20 mg/kg fluralaner, 0.025–0.050 mg/kg moxidectin, and 5–10 mg/kg pyrantel (as pyrantel pamoate) or 2.5–5.2 mg/kg afoxolaner and 0.5–1.0 mg/kg milbemycin oxime, respectively. Treatments were administered by clinic staff trained in study procedures. After the treatments were administered, each dog was observed for 10 min for any unfavorable incident related to treatment administration.

### Analysis

Efficacy of each product was determined by analysis of the per protocol population (PP) comprised of all dogs that were treated and examined according to the protocol. Any dog that received treatment was included in the assessment of product safety. Homogeneity of study groups at inclusion was evaluated descriptively. Means, standard deviations, minima, maxima, and medians were calculated for individual age, weight of the included dogs, and fecal egg count (FEC) of each nematode species before treatment. The comparison of FEC distributions by nematode species was restricted to those animals positive for the respective nematode at day 0. Frequency tables were used to compare the distribution of sex and breed in the two groups. The statistical analysis was performed by means of the software package SAS® (SAS Institute Inc., Cary, NC, USA, release 9.4).

Percentage efficacy was calculated by comparing the geometric mean FEC post-treatment with the pre-treatment geometric mean FEC in each treatment group using Abbott’s formula

$${\mathrm{Efficacy}}\,\,\% \,\, = \,\frac{{\overline{{\mathrm{X}}}_{{{\mathrm{PRE}}}} - \overline{{\mathrm{X}}}_{{{\mathrm{POST}}}} }}{{\overline{{\mathrm{X}}}_{{{\mathrm{PRE}}}} }}\,\,\,100$$

where X̅_PRE_ is the geometric mean of pre-treatment FEC and X̅_POST_ the geometric mean of post-treatment FEC.

Efficacy of the IVP against any intestinal nematode species was claimed if a FEC reduction in ≥ 10 dogs that were initially positive for that species was ≥ 90%, and if the geometric mean difference between pre- and post-treatment FEC was statistically significant (*α* ≤ 0.05) using a two-sided *t*-test for paired samples. To compensate for the skewed distribution, FEC were log-transformed and shifted prior to the statistical test: x_i_’ = log (x_i_ + 1). Efficacy was also calculated on the basis of arithmetic means and for geometric and arithmetic mean FEC reductions in the CP group.

Secondary efficacy was based upon the percentage of dogs that were nematode-free (no egg shedding) on day 14. The Farrington–Manning test of noninferiority for the risk difference was used with a level of significance of *α* = 0.025 (one-sided) and a tolerated difference of *δ* = 0.15 to compare the percentage of nematode-free cases in the IVP group with the percentage of nematode-free cases in the CP group [24]. Both the *P*-value and the lower 97.5% one-sided confidence limits were calculated. If the lower confidence limit was above −0.15, noninferiority was concluded. If the lower confidence limit was above 0, superiority was concluded.

## Results

Study dogs were enrolled at veterinary clinics in Albania (*n* = 60), Bulgaria (*n* = 111), Greece (*n* = 27), and Italy (*n* = 60) and treated and examined per protocol. No dogs were withdrawn during the study, and all completed cases could be included in the PP analysis.

Initial homogeneity of study groups, 172 dogs in the IVP group and 86 in the CP group, was demonstrated in the enrolled population, with no relevant differences between the study groups. Mean and median ages in the IVP group were 3.3 and 3.0 years, respectively, and in the CP group, 4.4 and 4.0 years. The age range in the IVP group was 0.25–12 years, and in the CP group, 1–12 years. Mean and median body weights were 19.3 and 19.1 kg in the IVP group, respectively, and in the CP group were 21.6 and 20.7 kg. Of the 113 females enrolled in the study, 19 (16.8%) were spayed, and of the 145 males, 11 (9.7%) had been neutered. More than 75% of enrolled dogs were described as mixed breed, with a range of other breeds represented in low proportions. The most common intestinal nematodes identified from enrollment samples were hookworms (168 dogs, 65.1% of all that were enrolled), *T. vulpis* (81 dogs, 31.4%), *T. canis* (61 dogs, 23.6%), and *T. leonina* (18 dogs, 7.0%).

### Safety assessment

There was no treatment-related AE reported in this study. Two dogs in the CP group were reported on days 12 and 14 to have tick infestations (infestations with ticks or fleas, or both were present in 134 (31.2%) of enrolled dogs, distributed across the two treatment groups). The tick infestations of these two animals resolved within 24 h, meeting the expected efficacy requirements for fleas and ticks of this product.

### Primary efficacy

On the basis of geometric mean reductions in FEC on day 14, relative to day 0, the efficacy of the IVP exceeded 99% against all intestinal nematode parasites that were detected prior to treatment in at least ten dogs (Table 1). The CP was also effective against intestinal nematode parasites, with FEC reductions from baseline exceeding 98%. Both treatments also appeared to be highly effective in eliminating capillarid-type eggs (capillarid genera were not differentiated). In the IVP group with sufficient dogs infected for protocol-allowance of statistical comparisons, geometric mean FEC reductions on day 14 versus day 0 were significant (*P* < 0.0001) for hookworms, *T. canis*, *T. leonina*, *T. vulpis*, and *Capillaria* spp. Using arithmetic means, the IVP was 95.5–100% effective in reducing FEC, and the CP was 90.3–100% effective.

**Table 1:** Geometric (arithmetic) means, percentage efficacy, and statistical comparison of fecal egg counts in dogs pre-treatment (administered on day 0) and 14 days post-treatment

	Hookworms	*Toxocara canis*	*Toxascaris leonina*	*Trichuris vulpis*	*Capillarid-type eggs*^*a*^
IVP (*N* = 172)	*n* = 110	*n* = 45	*n* = 11	*n* = 45	*n* = 10
Pre-treatment	219.0 (545.8)	223.5 (341.7)	124.4 (242.3)	130.9 (413.9)	74.0 (125.0)
Post-treatment	0.5 (24.8)	0.0 (0.0)	0.0 (0.0)	1.2 (9.3)	0.5 (5.0)
Efficacy (%)	99.8 (95.5)	100.0 (100.0)	100.0 (100.0)	99.1 (97.7)	99.4 (96.0)
*t*-Value	*t*_109_ = 25.04	*t*_44_ = 36.35	*t*_10_ = 12.86	*t*_44_ = 12.81	*t*_9_ = 8.35
*P*-value	< 0.0001	< 0.0001	< 0.0001	< 0.0001	< 0.0001
CP (*N* = 86)	*n* = 58	*n* = 16	*n* = 7	*n* = 36	*n* = 6
Pre-treatment	215.9 (471.6)	280.1 (455.0)	163.3 (321.4)	191.2 (362.8)	211.3 (266.7)
Post-treatment	3.1 (43.1)	0.7 (18.8)	0.0 (0.0)	0.1 (0.3)	0.9 (8.3)
Efficacy (%)	98.6 (90.9)	99.7 (95.9)	100.0 (100.0)	100.0 (99.9)	99.6 (96.9)

^a^Capillarid-type eggs not differentiated

*IVP* investigative veterinary product, *CP* control product, *NA* not applicable

Minimum dose, mg/kg: IVP fluralaner 10, moxidectin 0.025, pyrantel 5;

CP afoxolaner 2.5, milbemycin oxime 0.5

–Statistical comparisons based on geometric means, calculated for each species only if ≥ 10 dogs in a group had positive FECs at enrollment

### Secondary efficacy

Secondary efficacy results, based upon the percentage of dogs from which no nematode parasite ova were detected, were 87.8% and 72.1% of dogs free of parasite ova in the IVP and CP groups, respectively. With the lower confidence limit well above the noninferiority margin of −0.15, significant noninferiority of the IVP group in relation to the CP group was demonstrated (Table 2). As the lower confidence limit is even greater than 0, the percentage of nematode-free dogs in the IVP group was significantly superior (*P* < 0.025) to the result in the CP group.

**Table 2:** Noninferiority analysis (margin *δ* = 0.15): dogs in IVP treatment group free of nematode eggs 14 days post-treatment

	Risk difference	ASE	Z	Pr > Z
IVP, fluralaner–moxidectin–pyrantel	0.1570	0.0487	6.2974	< 0.0001

	Noninferiority limit	95% confidence limits	
IVP, fluralaner–moxidectin–pyrantel	−0.1500	0.0614	0.2525

*IVP* investigative veterinary product

*ASE* approximated standard error (Farrington–Manning)

*Z* Z-score (score statistic)

*Pr* Probability

## Discussion

Before treatment, the IVP group had ≥ 10 dogs that were infected with one or more of the following nematode species: hookworms, *T. canis, T. leonina*, and *T. vulpis*, resulting in a calculated efficacy of the IVP against these parasites of > 99%. Similarities in the eggs of *A. caninum* with those of *U. stenocephala* prevented their differentiation by mini-FLOTAC analysis alone [23, 25, 26]. Although *U. stenocephala* is believed to tolerate cooler climates, it is reported to be present throughout much of Europe, while *A. caninum* tolerates the warmer climates of the south [25–27]. Therefore, on the basis of the study site locations, it is likely that the ova classified in the study as hookworms were comprised of a mixed proportion of each of these two species. Importantly, separate laboratory studies have shown the fluralaner–moxidectin–pyrantel combination to be highly effective against both *A. caninum* and *U. stenocephala* [18], which the results of this study confirm.

In the current study, based on geometric mean FEC reductions, both IVP and CP were > 98% effective in the treatment of natural canine intestinal nematode parasite infections. Using arithmetic means, FEC reductions were different, in that the IVP (containing moxidectin and pyrantel) achieved efficacy > 95% against hookworms, but not the CP product (containing milbemycin oxime). This finding is consistent with early reports of limited milbemycin oxime efficacy against *A. caninum* and *U. stenocephala* when administered at 0.5 mg/kg as a single entity product [8, 28]. Furthermore, the significantly greater percentage of dogs in the IVP group with zero nematode eggs, relative to the CP group, suggests that moxidectin in combination with pyrantel is more effective than milbemycin oxime (0.5 mg/kg) against other intestinal nematode parasite species as well.

The absence of treatment-related adverse events in any study dog provides confirmation that the combination products used in the study retain the established safety profile of each active ingredient, established by decades of use in the treatment and prevention of internal and external parasites.

In addition, the complementary anthelminthic efficacy of pyrantel and moxidectin, with their different modes of action and pharmacodynamic properties in a fixed combination product, provides a broad spectrum of activity as well as a reduction of the risk of resistance development in canine nematodes.

## Conclusions

The orally administered fixed combination of fluralaner–moxidectin–pyrantel is safe and effective in the treatment of nematode parasite infections with hookworms, ascarids including *T. canis*, whipworm, and capillarids. On the basis of the complete elimination of parasite ova from the feces of treated dogs, this combination is significantly superior (*P* < 0.025) to an afoxolaner–milbemycin combination in the treatment of canine intestinal nematode parasite infections.

## Data Availability

All relevant supporting data have been included in the manuscript.

## Abbreviations

AE: Adverse event
CP: Control product
EPG: Eggs per gram
FEC: Fecal egg count
GCP: Good clinical practice
IVP: Investigational veterinary product
VICH: Veterinary International Conference on Harmonization
WAAVP: World Association for the Advancement of Veterinary Parasitology
